# *Candida albicans* gastrointestinal colonization resistance: a host-microbiome balancing act

**DOI:** 10.1128/iai.00610-24

**Published:** 2025-08-11

**Authors:** Derek J. Bays, Hannah P. Savage

**Affiliations:** 1 Department of Internal Medicine, Division of Infectious Diseases, School of Medicine, University of California Davis 12218, Sacramento, California, USA; 2 Department Medical Microbiology and Immunology, School of Veterinary Medicine, University of California Davis 70733, Davis, California, USA; University of California at Santa Cruz, Santa Cruz, California, USA

**Keywords:** *Candida albicans*, colonization resistance, microbiome, mycobiome

## Abstract

While *Candida albicans* is a common, commensal yeast colonizing 50%–60% of humans, it has the potential to expand in the gastrointestinal tract and enter the blood stream resulting in invasive candidiasis. Invasive candidiasis carries a mortality approaching 50%, especially in the most vulnerable, immunocompromised population. Antibacterial use causes an increase in *C. albicans* gastrointestinal colonization, indicating that the colonic microbiota plays a major role in preventing an uncontrolled expansion, a phenomenon known as colonization resistance. Antibacterials, medications, diet, and co-morbid conditions can all alter the microbiome, creating an altered environment known as dysbiosis. Our understanding of the microbiome continues to advance, and there is increasing evidence that the interactions that the microbiome has on the host are vital in maintaining colonization resistance to pathogens including *C. albicans*. This review will focus on colonization resistance to *C. albicans* within the gastrointestinal tract. The scope includes the benefits and consequences of *C. albicans* colonization, interkingdom interactions of the microbiome on *C. albicans*, microbiome-host interactions and how these modulate *C. albicans* colonization, and the impact of medications and diet on colonization resistance.

## INTRODUCTION

Despite fungi comprising only 0.1% of the microbes within the gastrointestinal microbiota (1), the importance of the mycobiome is increasingly recognized across several diseases beyond infections ranging from cancer (2) and inflammatory bowel disease (3). *Candida albicans* is a yeast that colonizes the human gastrointestinal tract in 50%–60% of adults (4). Under homeostatic conditions, it acts as a commensal opportunistic pathogen that can provide benefits to the host, including protection against *Clostridioides difficile* and other bacterial pathogens along with positive interactions in the immune system (5). Despite potential benefits to the host, there are many complications. *C. albicans* colonization is associated with exacerbation of inflammatory bowel disease (6) and serves as a reservoir for candidemia (*Candida* spp. in the bloodstream) in heavily immunocompromised patients, such as hematologic malignancy (7). Candidemia and invasive candidiasis carry a mortality approaching 50% (8), demonstrating the severity of this infection and importance of understanding the factors involved with *C. albicans* gastrointestinal colonization. For these reasons, in 2022, the World Health Organization designated *C. albicans* as a critical priority pathogen (9). Antibacterial use is a known risk factor for gastrointestinal colonization and expansion of *C. albicans* (4), arguing that the gastrointestinal microbiota serves a major function in protecting against colonization, a phenomenon known as colonization resistance (10, 11). However, as more information related to the microbiome is discovered, it is becoming clearer that the interactions within the microbiome, metabolites produced by the microbiota, and host-microbiome interactions often play a larger role than the individual species of the microbiota (12). In this review, we will briefly explore the benefits and consequences of *C. albicans* gastrointestinal colonization followed by an in-depth review regarding gastrointestinal *C. albicans* colonization resistance with a focus on microbiota-host-*C. albicans* interactions and common external factors such as antimicrobials and diet.

## BENEFITS AND COMPLICATIONS OF *C. ALBICANS* GASTROINTESTINAL COLONIZATION

This topic has been reviewed previously (5, 13) and will not be the main focus of this review. The clear complication of *C. albicans* colonization is related to the potential risk of development of candidemia. Koh et al. previously demonstrated that to develop invasive candidiasis from a gastrointestinal source in murine models, there needed to be immunosuppression (particularly neutropenia), gastrointestinal disruption, and gastrointestinal colonization of *C. albicans* (14). This demonstrates the major downside of gastrointestinal colonization in cases of hematologic malignancy, as immunosuppression and gastrointestinal disruption are unavoidable as part of cytotoxic chemotherapy. Relatedly, Zhai et al. showed that candidemia in adults receiving stem cell transplants is associated with an intestinal bloom of *Candida* spp. despite the use of antifungal prophylaxis (7). The same group went on to show that in *C. parapsilosis,* heteroresistance to antifungal prophylaxis can facilitate the expansion of *C. parapsilosis* in the presence of echinocandin prophylaxis (15). While it is unclear if this applies to all species of *Candida*, it highlights the limitations of our current antifungal prophylaxis preventative strategy for invasive candidiasis. This argues for the need to better understand the factors involved in gastrointestinal colonization of *Candida* spp. to identify novel prophylactic strategies.

Beyond the potential infectious complications of *C. albicans* gastrointestinal colonization, other negative effects have been described—most notably, the association between *C. albicans* gastrointestinal colonization and inflammatory bowel disease. Deficiency and polymorphisms in Dectin-1, the C-type lectin receptor for recognizing (1,3)-β-D glucans—a large component of *C. albicans* fungal cell wall— are associated with worse outcomes of ulcerative colitis (16). It is hypothesized that this loss of immune-mediated control of gastrointestinal colonization of *Candida* spp. and other fungi results in increased production of pro-inflammatory cytokines including interleukin (IL)−17 (16), which then exacerbates inflammatory bowel disease. Interestingly, after the use of IL-17 antagonists, there can be development of inflammatory bowel disease (17). This suggests that, under homeostasis, there is a balance of IL-17-mediated control of a gastrointestinal bloom of *C. albicans* without associated immune-mediated pathology (16, 18). When this is disrupted, there can be an expansion of *C. albicans*, an associated increase in IL-17, and accompanying immunopathology exacerbating inflammatory bowel disease (16).

Despite the negative associations with *C. albicans* gastrointestinal colonization, there are documented benefits. At least in murine models, pre-colonization of *Candida albicans* led to increased expression of IL-17A in gastrointestinal tissue with improved survivability in mice challenged with *Clostridioides difficile* compared to mice not pre-colonized with *C. albicans* (19). This seems to be achieved by *C. albicans* colonization promoting IL-17A, as treating mice with IL-17A and then infecting with *C. difficile* had similar survivability of mice pre-colonized with *C. albicans* (19). However, other work has demonstrated that *C. albicans* may help create an environment for *C. difficile* to thrive in (20). Additional *in vivo* studies are needed to confirm these findings. Human data are less clear if *C. albicans* colonization is protective against *C. difficile* colitis. In a prospective study evaluating patients with diarrhea being tested for *C. difficile* colitis, patients with *C. difficile* colitis were more likely to be colonized with *C. albicans* compared to patients without *C. difficile* colitis (21). However, another similarly conducted study demonstrated that *C. albicans* overgrowth in the gastrointestinal tract was seen more commonly in patients without *C. difficile* colitis (22). Given that *C. albicans* expands in the gastrointestinal tract in the setting of antibacterials (23, 24), it is possible that states of *C. albicans* overgrowth protect against *C. difficile* colonization due to niche preemption (25). Alternatively, *C. albicans* overgrowth may stimulate higher production of IL-17A (18), helping protect against *C. difficile* that is not conferred under lower levels of colonization. Additional studies are needed to determine the role of *C. albicans* gastrointestinal colonization and *C. difficile* outcomes, but regardless, there is compelling evidence in mouse models with biological plausibility on how *C. albicans* colonization helps prevent mortality from *C. difficile* colitis (19).

Beyond the protective role of *C. albicans* colonization in *C. difficile* colitis, there are other interkingdom interactions between *C. albicans* and bacteria in the gastrointestinal tract. In a neutropenic mouse model, *C. albicans* colonization reduced the virulence of *Pseudomonas aeruginosa* (26). *C. albicans* colonization inhibited expression of pyochelin and pyoverdine from *P. aeruginosa*, which are both involved in iron acquisition (26). That said, there is new work showing how *C. albicans* colonization can exacerbate *Salmonella* infection (27) by *Salmonella* directly altering *C. albicans* metabolism to increase arginine availability, demonstrating that interkingdom interactions are not always positive. Furthermore, a recent review by Wang et al. (28) highlighted six different interactions with varying benefits and consequences of *C. albicans* colonization (28) that will not be fully re-reviewed here. However, one that stands out is that in a post-antibacterial-depleted microbiota, *C. albicans* antagonizes *Lactobacillus* colonization and promotes *Enterococcus faecalis* (29), which is surprising given that others have shown that *E. faecalis* can produce a peptide, EntV, that has deleterious effects on *C. albicans* and reduced virulence of *C. albicans* in a *Caenorhabditis elegans* model (30). *C*. albicans can also utilize virulence factors to improve its ability to colonize the gastrointestinal tract when bacteria are present. In a recent landmark study by Liang et al., they showed that *C. albicans egf1* knockout strains, that are locked in yeast, form poorly colonized mice engrafted with bacteria (*Enterobacteriaceae coli, Klebsiella pneumoniae,* and *Enterococcus faecium*) (31). However, wild-type *C. albicans* can overcome this competition and maintain colonization. The authors went on to determine that candidalysin, a key virulence factor (32) only produced by hyphae, provided deleterious effects on bacteria including reducing metabolism and glucose utilization. Future studies are still needed to fully understand the different interkingdom interactions between *C. albicans* and the gut microbiota to better determine the benefits and complications of *C. albicans* gastrointestinal colonization.

## ANTIBACTERIALS AND *C. ALBICANS* GASTROINTESTINAL COLONIZATION

Antibacterials decrease colonization resistance to *C. albicans* in the gastrointestinal tract, allowing for an expansion (33) or colonization of mice, which typically have high colonization resistance against *C. albicans* (14). In a study investigating the effect of antibacterials in human cancer patients, there was a profound increase (as much as 1,000-fold increase) of gastrointestinal yeast after antibacterials (33). By exploring the function of the gastrointestinal microbiome in maintaining colonization resistance against *C. albicans*, it may be possible to identify novel ways to restore colonization resistance in the most vulnerable of populations that require antibacterials. There have been multiple approaches to better understand how antibacterials increase gastrointestinal colonization of *C. albicans*. Fan et al. demonstrated the importance of host defensins in maintaining colonization resistance against *C. albicans* in mice (24). They showed that antibacterials cause a reduction in Clostridial Firmicutes and Bacteroidetes that allows for *C. albicans* to colonize the gastrointestinal tract, and spontaneous recovery of the microbiota after stopping antibacterials is associated with a reduction in *C. albicans* (24). Importantly, this group did not stop looking at just the microbiota changes associated with *C. albicans* but went on to investigate the functional changes brought on by antibacterial-mediated microbiota depletion. They found that loss in *Bacteroides thetaiotaomicron* had a resulting decrease in *hif1a* expression, a decrease in the production of the antimicrobial peptide CRAMP in mouse colonocytes, and a corresponding increase in *C. albicans* (24). This study was the first, to our knowledge, to explore how the microbiota acts on the gastrointestinal epithelium to affect *C. albicans* gastrointestinal colonization through the production of antimicrobial peptides. Another antimicrobial peptide, Peptide YY, was shown to reduce *C. albicans* gastrointestinal colonization and virulence by Pierre et al. (34). Interestingly, the sensitivity to antimicrobial peptides may not apply to all strains of *C. albicans* (35). McDonough et al. demonstrated that different strains of *C. albicans* (529L and CHN1) can colonize the murine gastrointestinal tract without antibacterials, and that these strains were less susceptible to antimicrobial peptides compared to the standard lab strain of *C. albicans*, SC5314 (35). While antibacterials clearly increase the gastrointestinal colonization of *C. albicans*, this study demonstrated the importance of strain-to-strain variability in gastrointestinal colonization, which has been further explored with human isolates of *C. albicans* (36)

Guinan et al. investigated another potential host-microbiota interaction with *C. albicans* gastrointestinal colonization (37). They explored the impact on antibacterial treatment in mice and corresponding decreases in microbiota-derived short-chain fatty acids. They found that mice treated with antibacterials and infected via orogastric gavage with *C. albicans* had higher gastrointestinal colonization and lower short-chain fatty acids compared to mice not treated with antibacterials (37). Furthermore, they investigated the impact of short-chain fatty acids directly on *C. albicans in vitro* and showed that short-chain fatty acids inhibited *C. albicans* growth and germ-tube formation (37). However, this study did not evaluate the potential impact of short-chain fatty acids on the host.

In mouse models, antibacterials significantly deplete Clostridial Firmicutes and subsequently short-chain fatty acids, predominantly butyrate (38). Byndloss et al. showed that antibacterial-mediated depletion of *Clostridia* and *Clostridia-*produced butyrate had a profound impact on the host (38). They demonstrated that microbiota-produced butyrate is crucial in maintaining the hypoxic state of the colon through activation of PPAR-γ in the colonic epithelium (38). Maintenance of epithelial hypoxia was sufficient to prevent an expansion of *Escherichia coli* (38). Importantly, this group showed that microbiota replacement with *Clostridia* or butyrate supplementation could prevent an expansion of *E. coli* (38).

**Fig 1:**
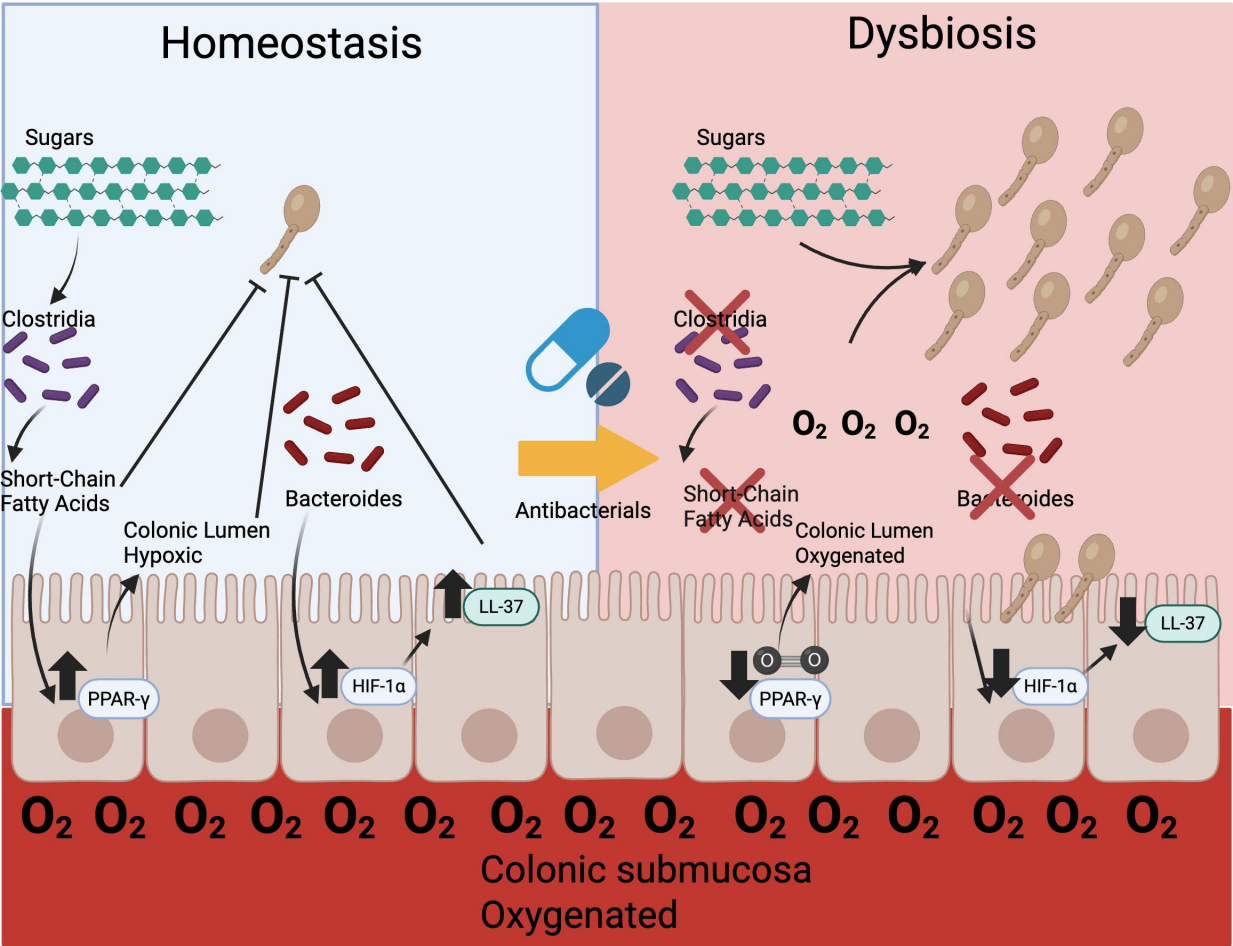
Under homeostatic conditions, there is colonization resistance to *C. albicans* mediated by several factors. The microbiota is composed predominantly of obligate anaerobes such as *Clostridia*, which ferments undigested sugars in the colon into short-chain fatty acids (butyrate) and limits nutrients for *C. albicans*. Butyrate then increases *Pparg* expression, promoting mitochondrial bioenergetics that consume oxygen from the submucosa, preventing oxygen from diffusing into the lumen of the colon and maintaining a hypoxic environment. Short-chain fatty acids also directly impair *C. albicans* growth and hyphal formation. *Bacteroides* also acts to increase HIF-1⍺, which promotes generation of the antimicrobial peptide LL-37, which directly inhibits *C. albicans*. Antimicrobials lead to disruption of the microbiota (dysbiosis) including loss of *Clostridia* and *Bacteroides*. Loss of short-chain fatty acid-produced *Clostridia* leads to an oxygenated epithelium allowing *C. albicans* to utilize carbon sources. Additionally, there is a loss of direct inhibition from short-chain fatty acids and LL-37. Collectively, these allow *C. albicans* to expand in the gastrointestinal tract and potentially become invasive.

Collectively, this work led our group to hypothesize that antibacterials may be reducing *C. albicans* colonization resistance through alterations in the colonic epithelium oxygen availability. We showed that in mice treated with antibacterials, there is a decrease in *Clostridia* and *Clostridia-*produced short-chain fatty acids with a corresponding increase in colonic epithelial oxygenation (23). Replacement of *Clostridia* was able to restore colonic epithelial hypoxia and reduce *C. albicans* colonization (23). Promisingly, we were able to use the PPAR-γ agonist, 5-aminosalicylic acid, to restore epithelial hypoxia and colonization resistance against *C. albicans* even in an antibacterial-depleted microbiota lacking short-chain fatty acids. While our group did not note significant differences in antimicrobial peptides as described previously, we did not assess if our mice were colonized with *Bacteroides thetaiotaomicron*, which could be a possible explanation. as there are known vivarium-vivarium microbiota differences (39–42). Based on the current body of evidence, it seems that antibacterials promote gastrointestinal colonization of *C. albicans* through multiple mechanisms (Fig. 1), but the unifying theme appears that there are microbiota-host interactions necessary to prevent the expansion of *C. albicans* in the gastrointestinal tract.

## BEYOND ANTIBACTERIALS AND *C. ALBICANS* GASTROINTESTINAL COLONIZATION RESISTANCE

While antibacterials are clearly linked to reducing gastrointestinal colonization resistance, there are additional studies demonstrating reduced colonization resistance independent of antibacterial use. Gastrointestinal inflammation lowered colonization resistance to *C. albicans* in a dextran sodium sulfate colitis model in mice (43). This group found that this was dependent on the presence of galectin-3 (43); however, other studies have shown that dextran sodium sulfate lowers the colonization resistance to *E. coli* by inducing oxygenation of the gastrointestinal epithelium (38), so it is possible that *C. albicans* also expands with dextran sodium sulfate colitis in an oxygen-dependent fashion (23). Interestingly, Panpetch et al. showed that *C. albicans* exacerbates dextran sodium sulfate colitis in mice, and *C. albicans* colonization and colitis could be reduced by pre-treatment with *Lactobacillus rhamnosus* (44). As *Lactobacillus rhamnosus* can produce short-chain fatty acids, including butyrate (45), it is possible that this is a similar mechanism as described by Byndloss et al. (38), but further studies would be needed.

Chemotherapy also disrupts the microbiota and alters colonization resistance to potential pathogens (46). In a study of human patients receiving chemotherapy for non-Hodgkin lymphoma, there were significant differences in the composition of the microbiota before and after chemotherapy without concurrent antimicrobials (47). Notably, there was a reduction in Firmicutes (containing *Clostridia*) and Actinobacteria with an increase in Proteobacteria (including *Enterobacteriaceae*) (47), which supports an environment where *C. albicans* could expand. This is consistent with another study (48) looking at microbiota changes in pediatric patients receiving treatment for acute myeloid leukemia, including reduction in *Bacteroides* spp. which can produce antimicrobial peptides to limit *C. albicans* colonization (24). However, they saw an expansion of *Enterococcus* spp., not *Enterobacteriaceae* (48), which may have been related to the antibacterial prophylaxis targeting gram-negative bacteria used in this study. While limited by having concurrent antibacterial use, this further supports the potential of chemotherapy to alter the microbiota and potentially support *C. albicans* colonization. The effects of chemotherapy on the mycobiome remain underexplored, but chemotherapy does seem to create microbiota disruption that would support *C. albicans* colonization with and without antibacterials, based on a reduction in *Clostridia* and *Bacteroides*. Furthermore, murine models with chemotherapy alone have demonstrated a change to the microbiota that could support *C. albicans* colonization through chemotherapy-induced depletion of microbiota-derived short-chain fatty acids (49). Zhai et al. demonstrated that during chemotherapy and antimicrobial prophylaxis, there is a loss of *Candida* spp. colonization resistance and an accompanying bloom of *Candida* spp. in the colon (7), and future studies could explore the effect of chemotherapy alone on colonization resistance.

The effects of non-antimicrobial and non-chemotherapeutic drugs on *C. albicans* are currently understudied; however, a study by Vich Vila et al. exploring microbiota changes associated with the use of 41 different commonly used drugs may provide some clues to their expected effects on *C. albicans* colonization (50). They analyzed metagenomic data from over 2,000 patients, either healthy controls, patients with IBS, or patients with IBD, and assessed changes in the overall microbial composition as well as changes in metabolic pathways and antibacterial resistance genes. Unsurprisingly, they found that antibacterial usage had a major impact on the microbiota, but perhaps more surprisingly, they found that proton pump inhibitors, metformin, and laxatives all caused significant changes as well. In particular, proton pump inhibitors had the largest number of associated changes in microbial taxa and metabolic pathways. However, this study only identified associations, not causal links, and the implications of these alterations for colonization resistance have yet to be determined. Whether these drugs cause a loss of colonization resistance similar to antibacterials is not currently known, but it may be worth investigating given the pervasiveness of these treatments.

## GASTROINTESTINAL METABOLITE PROFILE CHANGES AND *C. ALBICANS* COLONIZATION

Studies in this area are limited, but an examination of metabolite changes seen with antimicrobial therapy and diet alterations, two factors linked to *C. albicans* expansion, may provide clues about metabolite changes associated with *C. albicans* susceptibility. As antibacterials are a major risk factor for *C. albicans* colonization, it is important to consider the impact of antibacterials on the metabolite profile in the gastrointestinal tract. Antibacterials in mice reduce *Clostridia* with a corresponding increase in availability of carbohydrates, mainly sugar alcohols such as sorbitol (51). Others have also demonstrated that antibacterials in mice profoundly impact the intestinal metabolome, including an increase in carbohydrates (52). This decrease in *Clostridia* does cause a decrease in microbiota-derived short-chain fatty acids, resulting in an increase in colonic epithelial oxygenation, as discussed above (38). However, it also raises the question of the consequence of having increased carbon sources in the colon for potential pathogens, including *C. albicans,* to utilize. *C. albicans* readily uses glucose at sites of dissemination (blood, liver, and brain) where glucose is plentiful (53), but glucose is limited in the colon during homeostasis (54). We demonstrated that *C. albicans* predominantly reduces carbohydrates, including sugar alcohols such as sorbitol, when colonizing germ-free mice with *C. albicans* and comparing colonic metabolite profiles of germ-free mice or gnotobiotic mice colonized with *C. albicans* (23). While this work suggests that metabolite availability could impact colonization resistance, more work is needed to better understand the impact of antibacterial use, carbohydrate availability, and oxygen availability on *C. albicans* gastrointestinal colonization.

## IMPACT OF DIET ON *C. ALBICANS* COLONIZATION

Diet can have profound effects on colonization resistance to pathogens. Lee et al. demonstrated that a high-fat diet combined with antibacterials led to higher colonization with *E. coli* and a longer period of susceptibility to *E. coli* colonization compared to mice receiving antibacterials and a low-fat diet (55), supporting the impact of diet on colonization resistance. Others have investigated dietary changes on *C. albicans* colonization, indicating that this may be a modifiable factor when considering colonization resistance. Given that *C. albicans* prefers to utilize glucose when available (53), Weig et al. previously investigated the impact of carbohydrate supplementation in the diet on *C. albicans* colonization in healthy humans (56). However, their group did not find that carbohydrate supplementation altered *C. albicans* gastrointestinal colonization (56). Because the subjects of this study were healthy, they likely had an intact microbiota that could ferment excess carbohydrates not absorbed in the small intestine, potentially explaining their negative results (52). Another group looked at *C. albicans* in a cohort of healthy adults and only found that snacking seemed to correlate with increased *C. albicans* colonization and that high salt reduced it (57). While there has not been a substantial impact on *C. albicans* colonization in humans based on diet, it is worth emphasizing that these studies were done under homeostatic conditions where colonization resistance is high. Patients with conditions that may reduce colonization resistance (58), such as diabetes (59) or renal disease (60), are more likely to be placed on an altered diet (61, 62), so gaining a better understanding of the impact of diet on the expansion of opportunistic organisms may improve the safety and quality of life for many who live with these diseases.

Animal studies have also been performed to investigate this question. Yamaguchi et al. investigated the impact of a standard commercial mouse chow or a refined diet and showed that the refined diet eliminated colonization resistance, allowing for long-term colonization of *C. albicans* (63). This appeared to be due to a decrease in anaerobes and *Lactobacillus* spp. through an unclear mechanism (63). Future studies would need to be completed to determine which component of the refined diet was leading to an alteration in the microbiota that promoted *C. albicans* colonization, as this may be something to avoid in those at highest risk of invasive candidiasis. This finding has been supported by another group demonstrating how different standard mouse chows can alter the microbiota, including changes in *Clostridia*, further suggesting how diet impacts potential colonization resistance (64), but *C. albicans* was not studied. Another group demonstrated that a diet high in coconut oil had lower gastrointestinal *C. albicans* colonization (65). It was hypothesized that this may be due to fungicidal activity from medium-chain fatty acid production from coconut oil and by reducing long-chain fatty acids in the colon that *C. albicans* can use as a nutrient source (65). Additional studies are needed to determine what, if any, direct effects coconut oil supplementation has on the microbiota, along with evaluating if coconut oil supplementation reduces *C. albicans* in the gastrointestinal tract enough to reduce dissemination in an immunosuppressed model. Lastly, Fajstova et al. showed that mice treated with a high sugar diet in a dextran sodium sulfate colitis model had an unexpected colonization of *C. albicans* (66). However, it is unclear how much of this is due to a high sugar diet itself or brought on by inflammation, as the dextran sodium sulfate colitis model increases oxygen availability which can allow for expansion of *E. coli* (67).

While there is not enough data currently to support a specific diet when considering *C. albicans* gastrointestinal colonization, it likely warrants increased investigation given the murine studies and limitations of the human studies as discussed above. In particular, dietary factors that promote *C. albicans* colonization may also support colonization of other infectious organisms with similar metabolisms, such as other species of *Candida* or other opportunistic bacteria. Additionally, as patients with hematologic malignancy have a high risk of invasive candidiasis, it is important to note that these patients often receive an altered diet known as a neutropenic diet (68). The use of the neutropenic diet is controversial, as multiple meta-analyses concluded that there is no data to support the implementation of a neutropenic diet to prevent infections in the hematologic malignancy population (68, 69). Given evidence suggesting that there can be increased gastrointestinal colonization of *C. albicans* with different diets (63, 65), future studies should consider investigating if the neutropenic diet has any impact on *C. albicans* colonization.

## CONCLUSIONS

While there are potential benefits to *C. albicans* colonization within the gastrointestinal tract, the consequences of a gastrointestinal expansion of *C. albicans* in immunocompromised patients are clearly problematic, considering the potential for invasive candidiasis (7). Although low *C. albicans* colonization in healthy patients is potentially beneficial, antibacterial use and concurrent microbiota disruption set the stage for *C. albicans* colonization and expansion with many potential consequences. Unfortunately, the limitation of antimicrobials is often not feasible in those who are heavily immunocompromised, where antimicrobial prophylaxis not only reduces infections but also improves mortality (70). Additionally, despite antifungal prophylaxis, there are cases of breakthrough invasive candidiasis through drug resistance and host factors (8, 15). The collective work on colonization resistance (Fig. 1) demonstrates several microbiota-host interactions that are lost by antibacterials. Future studies should be completed to investigate how to **replace** these microbiota-host interactions with medications in environments where the microbiota is inevitably going to be altered by antibacterials and replacement of the microbiota may not be effective or safe.

It is worth emphasizing the limitation of this review focusing on *C. albicans*. This is mostly due to prior studies focusing on *C. albicans*, although there was a recent study looking at differences in colonization across several species of *Candida* (71), demonstrating the importance of consideration of different species for the future. Additionally, while *C. albicans* remains the most common species of invasive candidiasis, there are increasing rates of non-*albicans* infections (8). Lastly, many of the studies reviewed in this study used different strains of *C. albicans*. While SC5314 is considered the standard laboratory strain, there have been differences in colonization across different strains, including human-derived strains (35, 36). Another limitation to note is that this review focuses on gastrointestinal colonization resistance only. *C. albicans* can colonize the skin, oral cavity, and genitourinary tract as well (72), each of which has its own accompanying microbiota.

As *C. albicans* is a common commensal of humans, it is vital to understand the factors involved in the maintenance of colonization resistance due to the gastrointestinal tract serving as a reservoir for invasive disease. While there remain many unanswered questions, microbiota-host interactions seem necessary for maintaining colonization resistance, and future studies should focus on identifying these as potential targets for novel therapeutics.

As these studies demonstrate, the interactions between the host, the microbiota, and *C. albicans* are complex. During health, *C. albicans* can be a vital component of the mycobiota, but during disease, pharmaceutical administration, or potentially even with diet changes or in the context of certain genetic changes (73–75), *C. albicans* can become a pathogen. Together, a healthy microbiota and intestinal epithelium are major barriers for the development of invasive candidiasis, but if these components and their interactions go wrong, the gastrointestinal tract is also the site of *C. albicans* bloom prior to the development of invasive disease. It is vital that we gain an understanding of the factors involved in host-microbiota-*C. albicans* interactions to better understand the factors contributing to maintaining colonization resistance. Through this understanding, we can identify how signaling changes when colonization resistance fails. These signaling changes between the host and microbiota during the failure of colonization resistance will provide promising targets for novel therapeutics in the future.
